# GspD, The Type II Secretion System Secretin of *Leptospira*, Protects Hamsters against Lethal Infection with a Virulent *L. interrogans* Isolate

**DOI:** 10.3390/vaccines8040759

**Published:** 2020-12-14

**Authors:** Samantha Paulina Llanos Salinas, Luz Olivia Castillo Sánchez, Giselle Castañeda Miranda, Ernesto Armando Rodríguez Reyes, Liliana Ordoñez López, Rodrigo Mena Bañuelos, Luz Elena Alcaraz Sosa, María Guadalupe Núñez Carrera, Ramírez Ortega José Manuel, Carlos Alfredo Carmona Gasca, James Matsunaga, David A. Haake, Irma Eugenia Candanosa Aranda, Alejandro de la Peña-Moctezuma

**Affiliations:** 1 Teaching, Research and Extension Center for Animal Production in High Plateau, School of Veterinary Medicine and Zootechnics, National Autonomous University of Mexico, Queretaro 76795, Mexico; animal-spls@hotmail.com (S.P.L.S.); gicami.giss@gmail.com (G.C.M.); ieca@unam.mx (I.E.C.A.); 2 Unidad Académica de Medicina Veterinaria y Zootecnia, Universidad Autónoma de Nayarit, Tepic 63155, Mexico; olicastillos@yahoo.com.mx (L.O.C.S.); carmonagasca@yahoo.com.mx (C.A.C.G.); 3 División de Estudios de Posgrado, Facultad de Medicina, Universidad Nacional Autónoma de México, Coyoacán 04510, Mexico; ernestor.pcb@gmail.com; 4 Departamento de Microbiología e Inmunología, Facultad de Medicina Veterinaria y Zootecnia, Universidad Nacional Autónoma de México, Coyoacán 04510, Mexico; lilianaordonezlop@gmail.com (L.O.L.); mvz.rodrigo.mb@gmail.com (R.M.B.); jmro77@yahoo.com.mx (R.O.J.M.); 5 Departamento de Producción Agrícola y Animal, Universidad Autónoma Metropolitana, Tlalpan 14387, Mexico; luzelenaalcarazsosa@gmail.com; 6 Facultad de Medicina Veterinaria y Zootecnia, Benemérita Universidad Autónoma de Puebla, Centro Histórico 72000, Mexico; marychrist82@hotmail.com; 7 Veterans Affairs Greater Los Angeles Healthcare System, Los Angeles, CA 90073, USA; jamesm@ucla.edu (J.M.); dhaake@ucla.edu (D.A.H.)

**Keywords:** *Leptospira*, vaccine, recombinant, T2S, GspD, challenge

## Abstract

The wide variety of pathogenic *Leptospira* serovars and the weak protection offered by the available vaccines encourage the search for protective immunogens against leptospirosis. We found that the secretin GspD of the type II secretion system (T2S) of *Leptospira interrogans* serovar Canicola was highly conserved amongst pathogenic serovars and was expressed in vivo during infection, as shown by immunohistochemistry. Convalescent sera of hamsters, dogs, and cows showed the presence of IgG antibodies, recognizing a recombinant version of this protein expressed in *Escherichia coli* (rGspDLC) in Western blot assays. In a pilot vaccination study, a group of eight hamsters was immunized on days zero and 14 with 50 µg of rGspDLC mixed with Freund’s incomplete adjuvant (FIA). On day 28 of the study, 1,000 LD50 (Lethal Dose 50%) of a virulent strain of *Leptospira interrogans* serovar Canicola (LOCaS46) were inoculated by an intraoral submucosal route (IOSM). Seventy-five percent protection against disease (*p* = 0.017573, Fisher’s exact test) and 50% protection against infection were observed in this group of vaccinated hamsters. In contrast, 85% of non-vaccinated hamsters died six to nine days after the challenge. These results suggest the potential usefulness of the T2S secretin GspD of *Leptospira* as a protective recombinant vaccine against leptospirosis.

## 1. Introduction

Leptospirosis is a neglected and widespread zoonosis caused by the pathogenic species of *Leptospira*. Humans are accidental hosts who acquire the infection always from an animal source. Cattle, pigs, and dogs are the more commonly affected animal species with leptospirosis, causing abortion, infertility, and general systemic failure. However, rats, mice, and many other feral rodents are commonly infected with minor and unseen signs of disease; however, they are quite important reservoirs that spread, infecting leptospires in the environment through their urine. Rodents are considered as the main source of infection to human populations in rural and suburban areas with poor sanitary conditions [1,2]. More than one million human cases of leptospirosis and more than 50,000 deaths worldwide are reported to occur every year [3]. Human vaccines have been developed and administered to in risk populations only in China, Cuba, France, and Japan [4]. To date, the best way to prevent leptospirosis, besides the improvement of sanitary conditions, is animal vaccination. Currently available vaccines consist of cell suspensions of inactivated leptospires (bacterins), which have proved to be partially protective and limited to specific *Leptospira* serovars and not eliciting a long-lasting immunity. Immunity obtained with such a kind of vaccine is directed mainly towards leptospiral lipopolysaccharide (LPS), this being the main reason for their limited protection against disease caused by the specific serovars, those included in the bacterins formulation [5,6]. Research has been focused on conserved surface and subsurface leptospiral proteins as candidates for better vaccines against leptospirosis. Some relevant outer membrane proteins (OMPs), such as the porin OmpL1 and the lipoproteins LipL41 [7], LipL32 [8], LipL21 [9], Loa22 [10,11], and other OMPs like the Lig family of proteins A, B, and C [12,13,14], have been tested as vaccines, showing variable and often disappointing results [4]. Reverse vaccinology strategies (RV) have also been applied in the search for suitable protective antigens against leptospirosis without definitive results [15,16]. One functional protein that might be located on the *Leptospira’s* outer membrane is the secretin GspD, an important component of the type II secretion system (T2S). The OMP GspD is a protein known to be involved in toxin and enzyme secretion in Gram-negative bacteria [17]. In addition, GspD has been found as the twelfth most abundant component of the outer membrane (OM) proteome in *Leptospira* [18]. In the most complete RV approach so far performed to identify leptospiral vaccine candidates, GspD of *Leptospira borgpettersenii* serovar Hardjo strain L550 was used in a pool with four other OMPs. Despite a detectable antibody response, the protection was neither observed in this hamster model nor in 47 other protein pools tested in such a study [16]. Despite these results, we considered the GspD outstanding features, such as being a putative metabolically important protein, to be located on the surface of the outer membrane and so exposed to the immune system, and, finally, to be an abundant protein in *Leptospira*, to study GspD as a potential vaccine against leptospirosis. To determine the protection conferred against leptospirosis, we cloned and expressed the *gspD* gene of a virulent strain of *Leptospira interrogans* serovar Canicola (LOCaS46) into the pET-14b vector (Novagen™, Cambridge, MA, USA). The purified recombinant rGspDLC protein expressed in *E. coli,* when combined with incomplete Freund adjuvant (IFA) and administered twice in a group of eight hamsters, elicited protection against the infection with 1000 LD50 (lethal dose 50%), of a virulent homolog strain. Seventy-five percent of the challenged hamsters in the experimental group survived the infection (*p* = 0.021, Fisher’s exact test), and even 50% of them showed sterile immunity.

## 2. Materials and Methods

### 2.1. Genetic Procedures

#### 2.1.1. Construction of Recombinant Vectors

**Figure 1:**
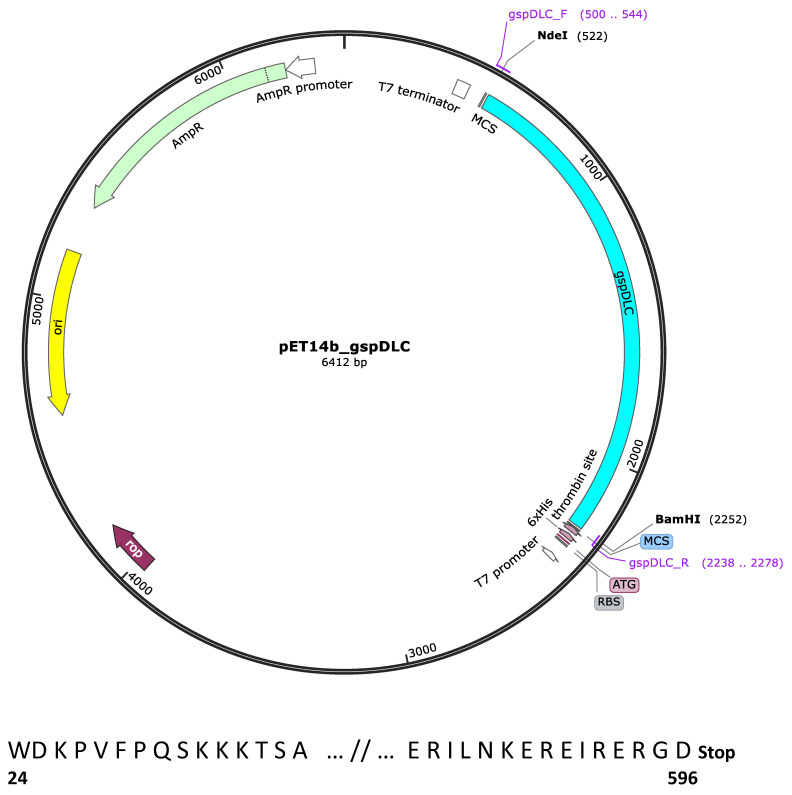
Map of the pET-14b_*gspDLC* recombinant expression vector. The 1734 bp *gspDLC* cloned insert is shown in pale blue color. *Nde*I and *Bam*HI restriction digestion cloning sites are shown. The map was built with the SnapGene™ 1.1.3 program. The fifteen first and last residues of the cloned peptide are shown, and numbers represent the actual numbered residue of GspD of *Leptospira interrogans* SV Canicola strain LOCaS46.

The *gsp* locus of the *L. interrogans* serovar Canicola strain LOCaS46 used in this work was sequenced. The *gspD* gene (Genbank accession number: MT743253) showed 100% identity with their homologs in several other strains of serovar Canicola available in the databases. Cloning of the *gspD* gene was performed using the procedure described by Pinne et al. [19], with some modifications. To remove the signal peptide, the 1734 bp PCR amplicon was designed to encode 573 residues of the GspD protein except for the first 23 aa. The DNA fragment was PCR-amplified from genomic DNA of the LOCaS46 strain, using the APM1f (GT GTT ACA TAT GGA CAA ACC AGT CTT T CC) and APM3r (TC ATT TCG GCC GCT CTT TCT CTG ATT TCT CTT TCT TTG) set of primers. These primers were designed with the inclusion of the appropriate *Nde*I and *Bam*HI restriction sites for cloning into the pET-14b expression vector (Novagen™, Cambridge, MA, USA). The diagram of the pET-14b_*gspDLC* construct was created with SnapGene™ (Figure 1). The PCR product size was verified by agarose gel electrophoresis, and the product was purified with the QIAquick PCR purification kit (Qiagen™, Redwood City, CA, USA), according to the manufacturer’s instructions. The DNA concentration was measured in a NanoDrop ND-1000 apparatus (Thermo Scientific™, Wilmington, DE, USA) at 260 nm. The ligation mixture was then chemically transformed into the *E. coli* DH5α cloning strain. The construct was sequenced (Instituto de Biotecnología, UNAM, Chamilpa, Cuernavaca) to verify that the insert was in the frame and subcloned into the BLR(DE3)pLysS *E. coli* expression strain (Sigma-Aldrich™, San Louis, MO, USA). The fifteen first and last residues of the rGspDLC protein are shown (Figure 1). Recombinant colonies, grown after incubation at 37 °C overnight on LB plates with ampicillin (100 µg/mL), were selected.

#### 2.1.2. GspDLC Expression, Screening, and Purification

Recombinant *E. coli* colonies were obtained by culture in selective LB-Amp plates (100 µg/mL) at 37 °C overnight and screened by PCR. Recombinants carrying the appropriate insert were sub-cultured in LB-Amp broth (100 µg/mL), shaking at 200 rpm and 18 °C until they reached an OD_600_ of 0.5. The cultures were then induced with 1 mM IPTG, incubating overnight for the expression of the rGspDLC protein. Cells were centrifuged at 6000× *g* for 3 min at 4 °C, and the lysis of the pellet was performed with the BugBuster reactive (Novagen™, San Diego, CA, USA), as the manufacturer’s instructions. The cell lysis reactive was mixed with 0.5 mM PMSF (Affimetrix™, Santa Clara, CA, USA) as proteases inhibitor and bovine pancreatic DNAse-I (Sigma™). The rGspDLC-His-tagged protein was detected in the *E. coli* cells extracts by Western blot assays and later purified by nickel affinity chromatography in Ni-NTA Superflow nickel charged columns (Qiagen™) with sodium phosphate and urea elution buffer, as specified by the manufacturer. The recombinant protein was concentrated by size exclusion using 30 kDa Amicon filter columns (Millipore™, San Louis, MO, USA) and centrifugation at 4000× *g*.

### 2.2. Antisera Production

New Zealand rabbits were immunized on days zero, 7, 14, and 21 with either 100 µg of the rGspDLC in PBS mixed with IFA or with a strain LOCaS46 culture and washed and concentrated up to 10^8^ leptospires/mL in PBS mixed with IFA. The rabbits were injected intramuscularly and bled to death on day 28 under ketamine dissociative anesthesia. The anti-rGspDLC polyclonal antiserum was evaluated in Western blot assays, and the anti-LOCaS46 antiserum was evaluated by the microscopic agglutination test (MAT).

### 2.3. Screening for GspD in Leptospira Serovars

OMP and total protein extracts of *Leptospira* cultures of serovars Australis, Ballum, Bratislava, Canicola, Hardjobovis, Grippotyphosa, Pomona, and Wolffi were obtained, as per the protocol described by Matsunaga [20]. These protein extracts were screened for the presence of GspD homologs in Western blot assays using the rabbit anti-rGspDLC polyclonal antiserum and a mouse monoclonal anti-rabbit IgG (Jackson ImmunoResearch™, West Grove, PA, USA) as the secondary antibody.

### 2.4. Leptospira Cultures and LD50 Calculation

An isolate obtained from an asymptomatic carrier dog was used for the challenging assays as well as for preparing the inactivated vaccine for the positive control group C. This isolate was characterized as *Leptospira interrogans* serovar Canicola by cross agglutination test (CAT), using a collection of serovars and specific rabbit antisera, and its identity was genetically confirmed by Multilocus Sequence typing (MLST) using the Ahmed et al. scheme [21], and such a strain was called LOCaS46. The LD50 of this strain was assessed in groups of eight Syrian hamsters in both intraperitoneal (IP) and intraoral submucosal inoculation (IOSM) [22]. This route of inoculation was attempted to get closer to the natural route of infection. To calculate the concentration of leptospires, 7-day old cultures incubated at 30 °C in Ellinghausen-McCullough-Johnson-Harris (EMJH) liquid medium [23] were ten-fold diluted in the same medium and quantified in triplicate in a Petroff-Hausser counting chamber (Hausser Scientific™). The LD50 was determined by the method of Reed and Müench [24]. Sub-cultures of leptospires were performed in both liquid EMJH and semisolid Fletcher media [25]. Fletcher medium was enriched with 8% of inactivated rabbit serum filter-sterilized at 0.22 µm.

### 2.5. Antigenicity of GspD in Infected Animals

Sera from five dogs, previously detected as *Leptospira* carriers by the culture of kidney tissues or urine samples, were used to search for specific anti-rGspDLC antibodies in Western blot assays. The dogs’ sera were previously shown to have a MAT titer equivalent to 1:1600 or higher against the serovars Canicola and/or Pyrogenes. Likewise, sera obtained from eight experimentally infected hamsters used to calculate the LD50 of the virulent strain LOCaS46 were tested in similar assays. The 15 µg of the rGspDLC protein was run in a 12% PAGE and transferred to a nitrocellulose membrane by electrophoresis. A 1:100 dilution of the primary antibody was used to recognize the transferred proteins, and a 1:400 dilution of an HRP-conjugate (as required, dog or hamster anti-IgG) was used as the secondary antibody (Jackson Immuno Research™, West Grove, PA, USA).

### 2.6. GspD In Situ Expression by Leptospira

To screen for the expression of GspD in tissues of hamsters experimentally infected with the virulent strain LOCaS46, the anti-rGspDLC and anti-LOCas46 rabbit polyclonal antisera were used in immunohistochemistry assays (IHC). Kidney and liver tissues obtained from the hamsters used in the LD50 assays were used for this experiment. A modification of the protocols, suggested by Massone et al. [26] and Haines and Chelack [27], was used as follows. Double serial dilutions of the anti-rGspDLC and anti-LOCaS46 antisera were prepared, from 1:25 as the initial dilution. Sections of each kidney and liver tissue samples were cut 3–5 µm thick using a Leica RM2245 microtome and mounted onto 3% poly-L-lysine-treated glass plates (Sigma™). Sections were dewaxed and washed twice for 30 min in xylol. Tissues were then hydrated 5 min thrice with decreasing concentrations of ethanol diluted in deionized water (96, 80, 70, 50%). To inhibit endogenous peroxidases, tissues were next treated for 30 min at room temperature with a 3.75% dilution of hydrogen peroxide in ethanol. Finally, tissues were washed for 5 min thrice with PBS, then for 20 min with a citrate buffer solution (10 mM sodium citrate, 0.05% Tween 20, pH 6.0), and again for 5 min thrice with PBS. For blocking unspecific reactions, the slides were covered with 60 µL of a 1:10 diluted goat nonimmune serum at 37 °C for 30 min in a humid chamber. After discarding the blocking serum, 60 µL of the rabbit polyclonal anti-rGspDLC or anti-LOCas46 antisera was applied in double serial dilutions from 1:25 up to 1:3200 in PBS pH 7.4 with 0.5% skimmed milk. Slides were incubated for 16 h at 4 °C in a humid chamber. After incubation, 5 min washes with PBS were done thrice, and then the slides were treated with 60 µL of biotinylated anti-rabbit IgG conjugate (Histostain-SP, Invitrogen™, Camarillo, CA, USA) and incubated at 37 °C for 60 min in a humid chamber. After three washes, 5 min each in PBS, 60 µL of the streptavidin-peroxidase conjugate (Histostain-SP, Invitrogen™, Camarillo, CA, USA) was added and incubated at 37 °C for 30 min in a humid chamber. For detection, the chromogen was prepared as follows: 50 µL of aminoethylcarbazol (Histostain-SP, Invitrogen™, Camarillo, CA, USA), 50 µL of the buffer provided by the manufacturer (Histostain-SP, Invitrogen™, Camarillo, CA, USA), and 50 µL of a 0.6% hydrogen peroxide solution in 850 µL deionized water; 60 µL of this chromogen solution was applied to the slides and incubated for 5 min. The reaction was stopped by rinsing in deionized water, and counterstaining was done for 5 min with Harris hematoxylin (Histostain-SP, Invitrogen™, Camarillo, CA, USA), and the slides were rinsed again in water and incubated for 1 min at 37 °C in PBS. Finally, slides were covered with Histomount resin (Histostain-SP, Invitrogen^®^, Camarillo, CA, USA) and a coverslip. Slides were observed under an AxioImager Z1 microscope (Carl Zeiss MicroImaging, Jena, Germany).

## 3. Vaccines

### 3.1. rGspDLC Vaccine

The purified rGspDLC protein was washed with PBS and concentrated to 1 µg/µL through a 30 kDa Amicon Ultra-4 filter column (Millipore Sigma™) in a final volume of 200 µL in PBS. Protein concentration was measured with the Micro BCA Protein Assay Kit (ThermoFisher™), according to the manufacturer’s instructions. The recombinant protein was used either alone (group A) or emulsified with IFA (Sigma™) in a 50% *v/v* suspension (group B) to get 50 µg of rGspDLC in a final volume of 100 µL.

### 3.2. Killed Cells Vaccine

The strain LOCaS46 of *L. interrogans* serovar Canicola was cultured in EMJH broth at 30 °C for 10 to 14 days. The cultures were concentrated by centrifugation and diluted in fresh EMJH medium to obtain a concentration of 10^9^ leptospires/mL. The concentration was estimated by counting the cells in a Petroff-Hausser counting chamber (Hausser Scientific™, Horsham, PA, USA). The leptospires suspension was inactivated with 0.05% formalin and emulsified with IFA (50% *v*/*v*).

### 3.3. Pilot Vaccination Study

Five groups of eight weeks old hamsters were treated as follows: group A, eight hamsters, vaccinated twice with 50 µg of the rGspDLC protein alone; group B, eight hamsters, vaccinated twice with 50 µg of the rGspDLC protein mixed 1:1 with IFA; group C, eight hamsters, vaccinated twice with a 10^9^/mL suspension of formalin-inactivated leptospires mixed 1:1 with IFA; group D, thirteen hamsters, injected twice with IFA, and group E, five hamsters, injected twice with sterile saline solution (Table 1). All treatments were freshly prepared in a final volume of 100 µL per hamster and injected subcutaneously on days zero and 14 of the experiment. On day 28, groups A to E were infected with the virulent *Leptospira interrogans* serovar Canicola strain LOCaS46, using an inoculation route by intraoral submucosal injection (IOSM). A dose of 1000 LD50 (40,000 leptospires) in a volume of 100 µL was applied with a 31G needle under isoflurane anesthesia, as previously described by Asoh et al. [22]. The IOSM route was selected to achieve a closer to the natural route of infection for leptospirosis when compared with the commonly used IP route of infection. An additional group of three non-infected hamsters was used as a control. The hamsters were kept in a 10/14 h light/darkness ratio during the length of the study, and food and clean water were supplied ad libitum. A final point of the experiment was established as the loss of 10% corporal weight to avoid unnecessary suffering of the infected specimens. Hamsters were observed daily and weighed to sense such an established final point. If they were not found dead, when the final point was reached or at the end of the experiment on day 56, the hamsters were killed by an isoflurane anesthesia overdose and necropsy. All animals were observed for 3 min after isoflurane overdose to confirm the death before proceeding to necropsy. During necropsy, kidney, liver, and urine samples were aseptically obtained for culture in EMJH and Fletcher media for PCR and histopathology and sera samples to screen for anti-*Leptospira* MAT antibodies. A direct macerate of kidney and liver tissues and blood as well as urine samples (when possible) was diluted directly into EMJH medium and observed under dark-field microscopy to assess for the presence of leptospires (X800 magnification).

### 3.4. Histopathology

A complete kidney and 0.5 cm^3^ of liver were obtained at necropsy, and the samples were immediately fixed in a 10% buffered formaldehyde solution, routinely processed with paraffin, and consecutively sectioned at 5 µm. For microscopic examination and scoring, some sections were stained with hematoxylin-eosin, and others were processed for immunohistochemistry, as previously described. The histomorphological evaluation of kidney lesions—indicative of *Leptospira* infection—tubular cortical necrosis, interstitial hemorrhage, tubular epithelium degeneration, intratubular hyaline eosinophilic content (tubular proteinoses), thrombosis, neutrophilic tubulitis, and interstitial inflammation, mainly composed of neutrophils, lymphocytes, and macrophages, was performed. The evaluated hepatic lesions were interstitial hemorrhage, vacuolar degeneration, necrosis, thrombosis, and interstitial inflammation, mainly composed of neutrophils, lymphocytes, and macrophages. The criteria for the histopathology evaluation was rated from zero to 4 according to the severity of the lesions: 0 as normal (0% coverage), 1 as minimal (up to 10% coverage), 2 as mild (up to 25% coverage), 3 as moderate (up to 50% coverage), and 4 as severe (more than 50% coverage). The sum of each lesion score per hamster in a group was finally expressed as an average. Slides were examined under an AxioImager Z1 microscope (Carl Zeiss MicroImaging, Jena, Germany) for histology or under dark-field microscopy to screen for leptospires in tissues.

## 4. Statistical Analysis

Where appropriate, the data are presented as means and standard deviations of the means. Comparisons of the severity of the renal lesions and the invasion of *Leptospira* in different tissues among hamster groups A to E were performed using the Student’s *t*-test and by the one-way ANOVA calculator; comparisons of the survivals in groups A to D were performed using the Fisher’s exact test; a *p*-value ≤ 0.05 was taken as statistically significant.

### Ethical Approval

All animal procedures in this study were performed according to the Mexican Official Standard NOM-062-ZOO-1999 (Technical Specifications for the Production, Care, and Use of Laboratory Animals) and the NOM-033-SAG/ZOO-1995 (Methods to Kill Domestic and Wild Animals) and were approved by the Institutional Committee for Care and Use of Experimental Animals of the School of Veterinary Medicine of the National Autonomous University of Mexico (CICUAE-FMVZ/UNAM), under the registration number: MC2014-27.

## 5. Results

### 5.1. Expression of the rGspDLC Protein and Antisera Production

**Figure 2:**
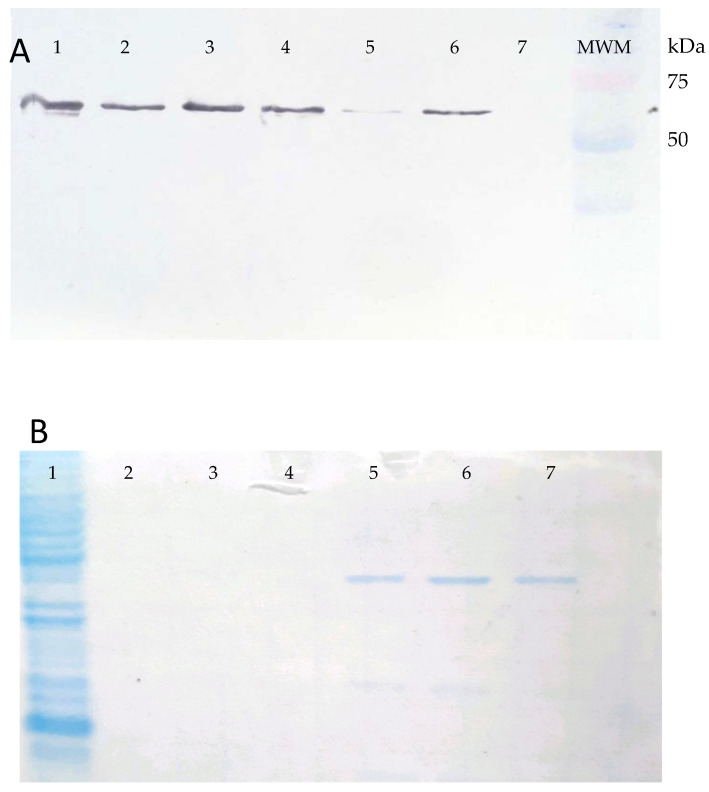
Panel (**A**). Western blot of the BL21(DE3) pLysS (Promega™) *E. coli* cells transformed with the pET-14b_gspDLC recombinant expression vector and induced with 1 mM IPTG (lanes 1–6). Lane 7, uninduced control. An approximately 63 kDa band was observed corresponding to the rGspDLC protein reactive to an anti-His monoclonal antibody. Panel (**B**). Coomassie blue-stained acrylamide gel, showing the rGspDLC purified by Ni-affinity chromatography and sodium phosphate/urea elution buffer. Lane 1, recombinant *E. coli* lysate; lanes 5, 6, and 7 show the purified recombinant protein.

The 1734 bp DNA fragment of the *gspD* gene of *Leptospira interrogans* serovar Canicola strain LOCaS46, cloned into the pET-14b (Novagen™ Cambridge, MA, USA) expression vector and chemically transformed into the BL21(DE3) pLysS (Sigma-Aldrich™, San Louis, MO, USA) *E. coli* cells, expressed the predicted 63 kDa poly-His-tagged protein when induced with IPTG, as described (Figure 2A).

The protein yield of the recombinant GspD secretin (rGspDLC) was approximately 80 to 120 µg/L of culture. The rGspDLC protein was purified by Ni-affinity chromatography and concentrated by size exclusion up to 1 mg/mL using 30 kDa Amicon filter columns (Millipore™), and an estimated 60 to 70% purity of the recombinant protein was estimated based on Coomassie blue-stained acrylamide gels (Figure 2B). The anti-rGspDLC rabbit antiserum reached a titer of 1:32,000 in Western blot assays. On the other hand, antiserum obtained from rabbits immunized four times with a suspension of the heat-inactivated *L. interrogans* serovar Canicola strain LOCaS46 reached a 1:12,800 antibody titer in the MAT (data not shown).

### 5.2. GspD in Leptospira Serovars, Antigenicity, and In Situ Expression

**Figure 3:**
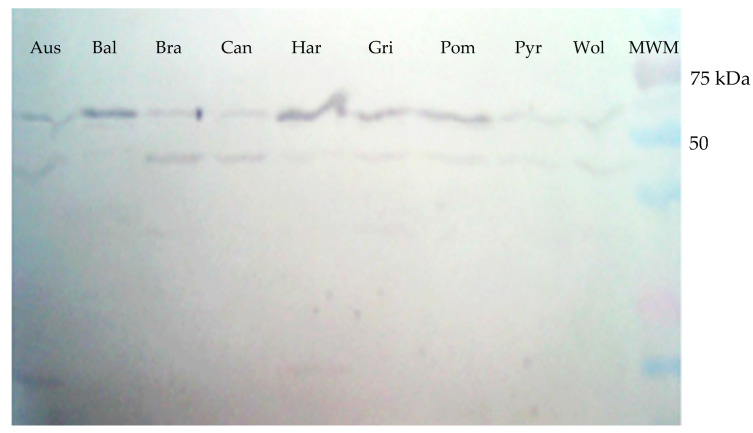
Western blot of the total protein extracts obtained from cultures of nine *Leptospira* serovars. An approximately 63 kDa band was shown when a rabbit antiserum against rGspDLC was used in a 1:100 dilution. Aus: Australis; Bal: Ballum; Bra: Bratislava; Can: Canicola; Har: Hardjoprajitno; Gri: Grippotyphosa; Pom: Pomona; Pyr: Pyrogenes; Wol: Wolffi.

A protein of approximately 63 kDa was detected in the extracts of all nine *Leptospira* serovars tested when the polyclonal rabbit antiserum raised against the rGspDLC protein was used in Western blot assays (Figure 3). An additional band of approximately 45 kDa was also observed in such assays in most of the *Leptospira* serovars. This finding was maybe due to the presence of an undetermined leptospiral protein with a structural region similar to rGspDLC, which was recognized by the polyclonal rabbit antibody.

**Figure 4:**
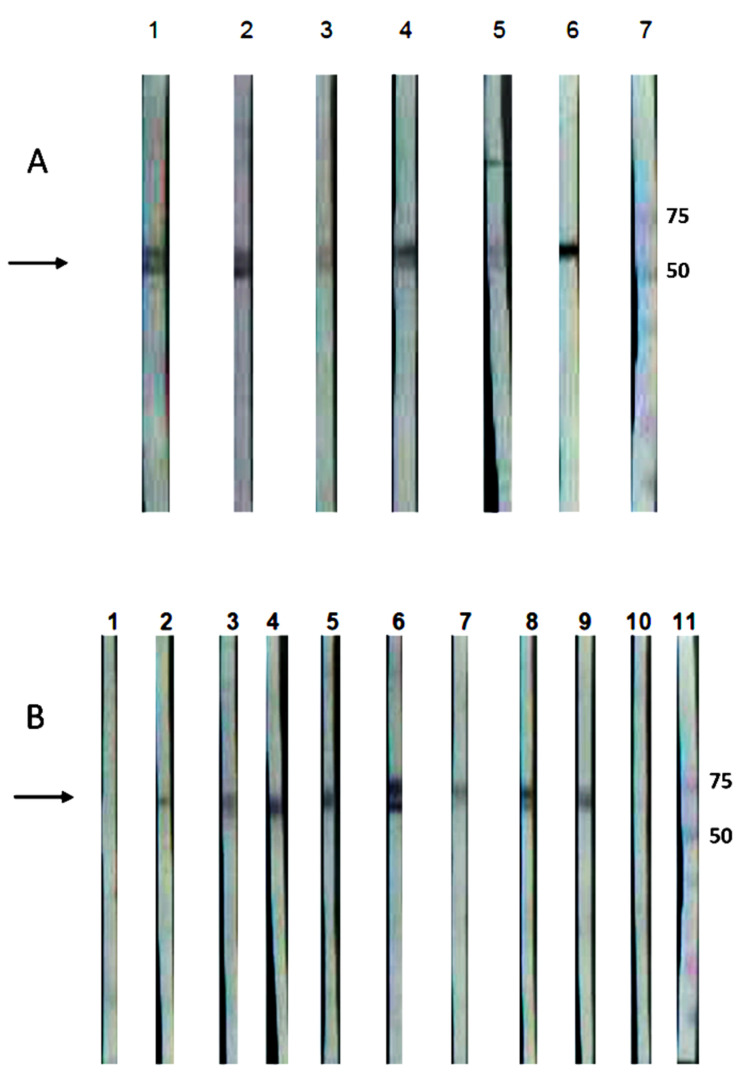
Western blot of the recombinant GspD secretin of *Leptospira interrogans* serovar Canicola (rGspDLC), expressed by *E. coli* BL21(DE3)pLysS transformants. Panel (**A**): Detection of an approximately 63 kDa band (arrow), using five sera from four naturally infected dogs (lanes 1–4) and one convalescent dog with leptospirosis (lane 5). Infection with serovar Canicola was confirmed by culture and MLST. Lane 6, anti-His-HRP positive control; lane 7, molecular weight marker. Panel (**B**): Detection of an approximately 63 kDa band (arrow), using the sera from eight hamsters experimentally infected with the virulent serovar Canicola strain LOCaS46 (lanes 2–9), and the infection was confirmed by culture. Sera from two non-infected hamsters were used as negative controls (lanes 1 and 10), where a band was not detected; lane 11, molecular weight marker.

Similarly, the sera from five dogs, previously identified as *Leptospira interrogans* serovar Canicola carriers, and the sera from the experimentally infected hamsters in this work showed IgG antibodies, detecting the 63 kDa band in Western blots of the rGspDLC protein expressed by the *E. coli* transformants (Figure 4).

**Figure 5:**
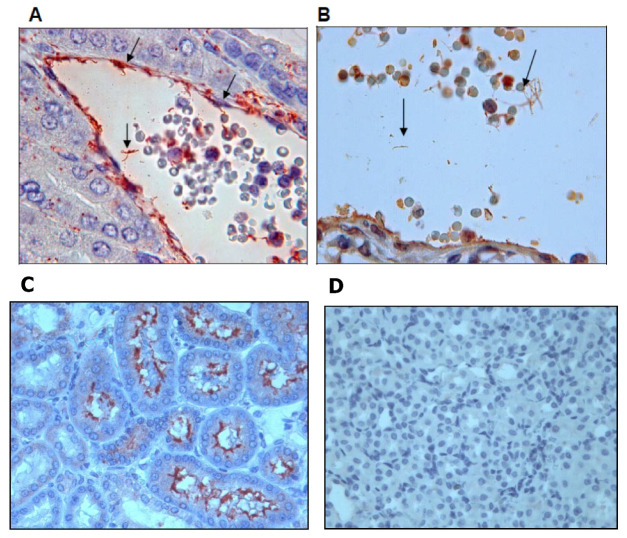
Immunohistochemistry assay on renal tissue of experimentally infected hamsters (*Mesocricetus auratus*). Infection was performed by intraperitoneal inoculation with the virulent strain LOCaS46 of *Leptospira interrogans* serovar Canicola. Panels (**A**,**C**): Detection of leptospires using a 1:200 dilution of rabbit polyclonal hyperimmune antiserum against *L. interrogans* serovar Canicola. Panel (**B**): Detection of leptospires using a 1:25 dilution of the rabbit polyclonal antibody against the recombinant GspD secretin of *L. interrogans* serovar Canicola (rGspDLC). Panel (**D**): Renal tissue of a non-infected hamster used as a control.

Further IHC assays using a 1:25 dilution of the anti-rGspDLC rabbit polyclonal antiserum detected leptospires in kidney tissue of the experimentally infected hamsters (Figure 5B), and a rabbit polyclonal antiserum against serovar Canicola (anti-LOCaS46) was used as a positive control in such IHC assays (Figure 5A,C). The specificity of both antisera (anti-rGspDLC and anti-LOCaS46) was proved in renal tissue of non-infected hamsters in similar assays that showed no unspecific reactions (Figure 5D).

### 5.3. Assessment of the Immunogenicity of the rGspDLC Protein

Results of the pilot vaccination study are shown in Table 1. The LD50 for Syrian hamsters of the virulent strain LOCaS46 of *L. interrogans* serovar Canicola was determined to be as low as 4 leptospires in the IP route and 40 leptospires in the IOSM route. Infection rates were determined by PCR and culture. No infection could be determined in seven out of eight hamsters in group C immunized with the homologous bacterin used as a positive control. Encouraging results were observed in the hamsters in group B vaccinated with the rGspDLC-IFA mixture, where a 75% survival was observed, and additionally, three out of the six hamsters that survived were shown to be free of *Leptospira* infection by culture and PCR (sterile immunity). However, for the hamsters in group A vaccinated with the rGspDLC alone, all of them were shown to be infected regardless of a 65% protection conferred against disease. The negative control group D treated exclusively with IFA showed only a 15% survival (85% lethality; *p* = 1.000, Fisher’s exact test). In addition, no survivals were observed in the control group E (Saline, *n* = 5), confirming, in these two control groups, the high virulence of the challenging strain LOCaS46 used in the pilot vaccination study.

### 5.4. Infection Assessment by Culture and PCR

All hamsters that lost 10% of body weight, established as the final point of the experiment, were either euthanized by an isoflurane overdose or found dead at days 9 or 10 after the challenge. General moderate jaundice was observed in most animals. At necropsy, kidneys and liver showed moderate congestion, and evident hemorrhages were observed in the lungs. The rates of infection assessed by culture and PCR were as high as 100% in the control groups D (IFA) and E (saline) and also in the hamsters of experimental group A (rGspDLC alone), despite the 65% protection against disease observed in this group. In contrast, three out of eight hamsters in group B (rGspDLC + IFA) (37.5%) and seven out of eight hamsters (87.5%) of group C (bacterin) showed no evidence of infection either by culture or PCR (Table 1). Statistically significant lowest infection rates were observed in hamsters of group C (bacterin) (*p* < 0.0001). Groups A and B also showed statistically significant lower rates of infection when compared with the control group D (IFA); however, there was no statistically significant difference between those two groups (*p* > 0.5).

### 5.5. Infection Assessment by Histopathology

**Figure 6:**
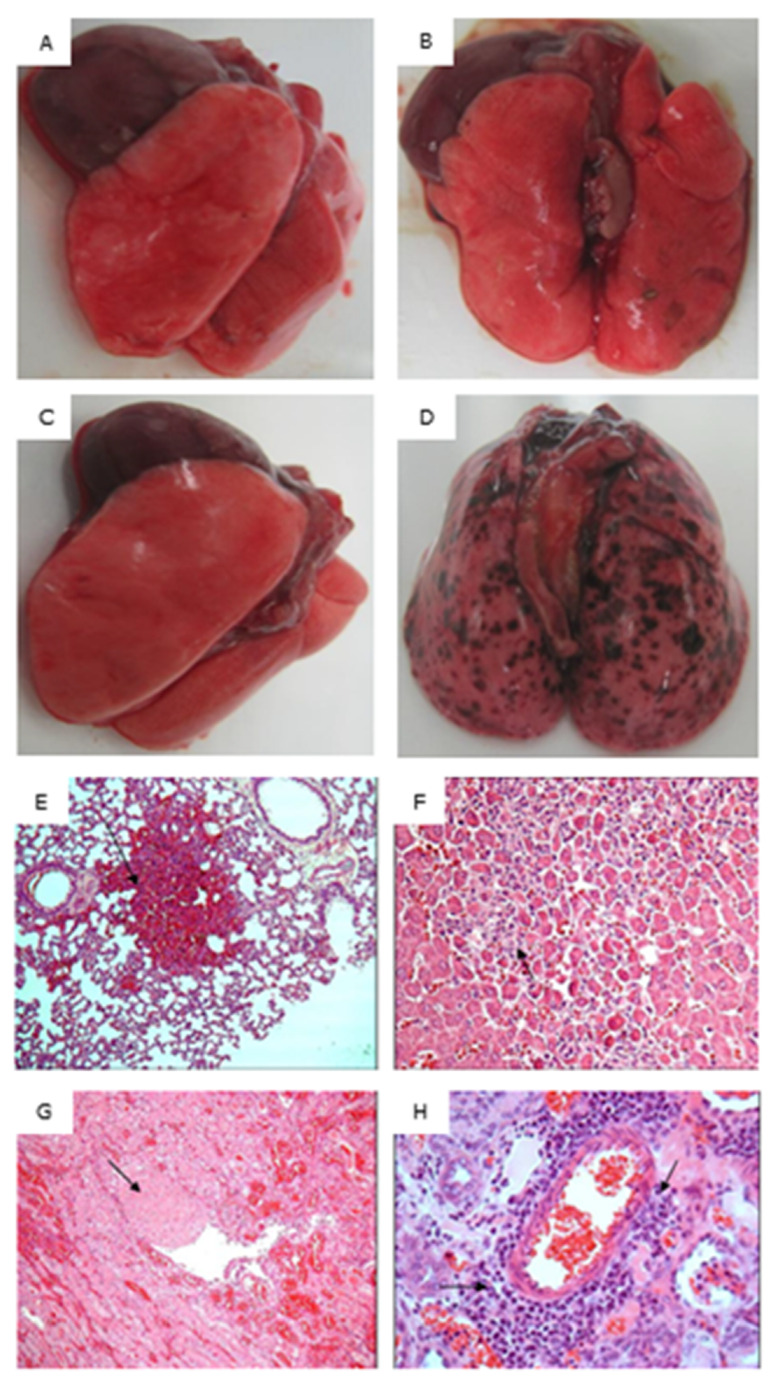
Pathology assessment of lungs from experimentally infected hamsters (Panels (**A**–**D**)), and HE 100X histopathology of the lung (Panel (**E**)), liver (Panel (**F**)), and kidney (Panels (**G,H**)) of a hamster in the negative control group D (IFA). Panel (**A**): Lung of a survival hamster in group A (rGspDLC alone); Panel (**B**): Lung of a survival hamster in group B (rGspDLC-IFA); Panel (**C**): Lung of a survival hamster in group C (serovar Canicola bacterin); Panel (**D**): Lung of a dead hamster in group D (IFA); Panel (**E**): Pulmonary hemorrhage; Panel F: Hepatocellular degeneration and necrosis and inflammatory infiltration; Panel (**G**): Renal vascular thrombosis; (**H**): Renal perivascular inflammatory infiltration, mainly composed of lymphocytes and plasmatic cells.

Experimental infection with the virulent *L. interrogans* serovar Canicola strain LOCaS46 produced macroscopic lesions characteristic of active leptospirosis, such as general jaundice, pulmonary hemorrhages (Figure 6A–D), and congestion in kidneys and liver. Microscopic lesions included tubular cortical necrosis, interstitial hemorrhage, tubular epithelium degeneration, intratubular hyaline eosinophilic content, thrombosis, neutrophilic tubulitis, and interstitial inflammation, mainly composed of neutrophils, lymphocytes, and macrophages (Figure 6E–H). The lowest average scores for renal lesions were observed in group C (bacterin) (mean = 3.5; *p* = < 0.0001). Meanwhile, the experimental groups A (rGspDLC alone) and B (rGspDLC-IFA) showed similar renal lesions scores (group A: mean = 4.875; *p* = 0.003026) (group B: mean = 5; *p* = 0.010007). The *p*-value of the infection assessment was calculated by comparison with the control group D (IFA), which showed the highest score (mean = 7.75). Results were statistically significant in the one-tailed t-test calculator at a significant level of 0.05 (Table 1).

## 6. Discussion

We report here that a recombinant form of the GspD protein of *Leptospira interrogans* serovar Canicola (rGspDLC) expressed in *E. coli* offered a 75% protection against disease in hamsters challenged with 1000 LD50 of a virulent homolog strain (*p* = 0.021, Fisher’s exact test). Although the minimal 80% protection established by the World Organization for Animal Health (OIE) was not achieved, the results are encouraging in the light that in addition to protection against disease, the experimental group B (rGspDLC-IFA) showed that three out of the six hamsters that survived had no evidence of infection either by culture or PCR from urine, liver, or kidney samples (Table 1). Protection was also inferred by the lesions observed at necropsy. Rates of the lesions observed in kidneys and liver in the experimental group B (mean = 5) were statistically significantly lower than the rates in control group D (IFA) (mean = 7.75) (*p* < 0.02). Besides, despite that group C (bacterin) showed the lowest rate of lesions, there was no statistically significant difference in the lesions rate between this and the two experimental groups A and B, as was analyzed by the one-way ANOVA calculator (*p* > 0.05) (Table 1).

The IP route of infection has been widely used for testing the efficiency of vaccines against leptospirosis, despite not being the natural route of infection [4,28]. In our experimental vaccination assay, we tested the previously described IOSM route of infection [22] applied under isoflurane anesthesia; the aim of the method was to mimic a closer to the natural route of infection. The LD50 for hamsters of the challenging strain *L. interrogans* serovar Canicola LOCaS46, using the IOSM route, was equivalent to 40 leptospires; this dose was four times higher than the lethal dose observed for serovar Losbanos strain K37, using the same infection route [22]. On the other hand, the IOSM LD50 of strain LOCaS46 was ten times higher than the IP route of infection for the same strain (data not shown) but still was very suitable for the experimental infection assays. Coutinho et al. tested the subcutaneous (SC) and intradermal (ID) routes of infection in a study to compare the kinetics of leptospiral infection. A dose of 2 × 10^6^ of *L. interrogans* serovar Copenhageni strain Fiocruz L1-130 was used, and only a slight difference in the burden of infection in the kidney was found among both routes of infection, reported at days 3 to 4 for the ID route and at days 4 to 5 for the SC route. The IP route was not tested in such a study [28].

**Figure 7:**
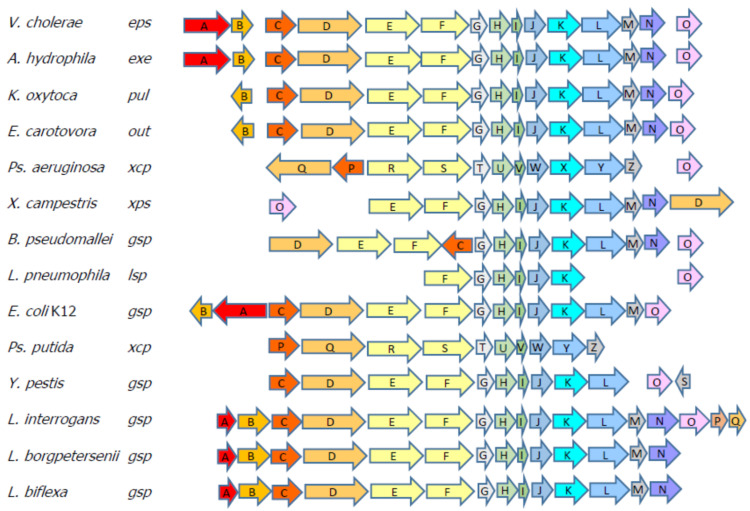
Schematic of the type II secretion system (T2S) loci in different bacteria. *L. interrogans* shows 17 ORFs in the *gsp* locus; meanwhile, *L. borpetersenii* and *L. biflexa* both show only 14 ORFs in their loci. (Modified from Sandkvist, 2001 [38]).

Surface proteins act as potential protective antigens due to their direct interaction with the cells of the host, and so both pathogenesis and immune response develop in parallel [29]. In *Leptospira,* surface and subsurface proteins, such as LipL32, LipL21, LipL41 [7,9,30], and others like OmpL1, LemA, LigA, LigB [13,31,32], have been shown to be localized in the *Leptospira* surfaceome and to be conserved among pathogenic serovars; for these reasons, they have been considered as candidates to develop wide spectrum vaccines that may offer heterologous immunity [20,33,34,35]. The secretin subcomplex of the type II secretion system (T2S) has been shown to be built as a pentadecamer of the GspD protein, and that arrangement provides a pore through to the outer membrane in the *Proteobacteria* [17]. Sequencing of *Leptospira* genomes has revealed a T1S and T2S but no other secretion systems, with complete Sec and TAT translocases systems [36]. We have shown that the GspD protein of the T2S is expressed by every *Leptospira* serovar that was screened in this work, as shown in the Western blot using an anti-rGspDLC polyclonal antibody (Figure 3). The detection of an additional 45 kDa band in most of the serovars tested in the assay (all of them but Ballum) might be the result of an undetermined immunoreactive leptospiral protein with a similar structural region that was detected by the polyclonal rabbit antisera. On the other hand, we also showed that GspDLC might well be a surface protein, as shown by the immunohistochemistry assays (Figure 5). Bioinformatic comparison of Gram-negative genomes revealed that some of the T2S genes are common but not universally conserved. Bacteria, such as *Klebsiella, Pseudomonas, Vibrio,* and *Aeromonas*, have been used as models for the study of the T2S components and the understanding of their secretion mechanisms [37,38]. While highly prevalent in γ– and β–proteobacteria, the T2S is also recognized in members of the δ and α classes [17,39]. About 12 to 15 different components have been determined as part of the T2S in the Gram-negative cell envelope, spanning from the cytoplasmic membrane to the outer membrane [40]. The first insight of the presence of a T2S in *Leptospira* has been revealed by MALDI-TOF mass spectrometry of Triton X-114 outer membrane extracts of *L. interrogans* serovar Lai grown at 20, 30, and 37 C. The analysis has identified, amongst others, the presence of pL18, an 18 kDa protein that is expressed under all tested conditions [33]. The in silico analysis of the predicted encoding sequence of 14 residues of the pL18 protein has shown significant similarity with GspG, a pseudopilin of the T2S in *E. coli, Pseudomonas,* and other Gram-negative bacteria [41,42,43,44]. Further chromosome walking sequencing has found a series of 14 ORFs corresponding to homologs of the T2S components in the serovar Hardjobovis L550 genome [45]. In *Leptospira,* 17 genes encoding Gsp components are found in serovars Lai and Copenhageni [46,47], but only 14 homologs are present in serovar Hardjobovis and in the nonpathogenic serovar Patoc [45,48]. Despite that *Leptospira biflexa* also harbors an encoded T2S locus, their genes show significant divergence when compared with those of the pathogenic *L. interrogans* or *L. borgpetersenii* serovars, with a Clustal W general identity of 59% amongst their *gspD* genes. The *gsp* loci in *Leptospira* show a similar order of genes when compared with Gram-negative bacteria [38] (Figure 7).

The T2S in Gram-negative bacteria is considered the main secretion system involved in delivering a variety of toxins and enzymes to the outer surface or the extracellular space. Such secreted products include acyltransferases, amylases, kinases, celluloses, pectinases, ADP-ribosylation enzymes, proteases, lipases, and phosphatases, which break down complex biochemical components, thus conferring survival advantages to both pathogenic as well as environmental bacteria [17,49,50]. It has been shown that *Leptospira* synthetizes diverse hydrolytic enzymes, such as hyaluronidase [51], catalase [52], lipases [53], putative cytochrome oxidases [54,55], and others like the virulence modifying proteins (VMPs) [56] and the putative Quorum sensing (QS) proteins [57], which might well be secreted by the leptospiral T2S. The presence of a T2S in *Leptospira* implies its role in metabolism, but it also might well be related to pathogenesis, as it has been reported for other Gram-negatives, such as the *Vibrio cholerae* CLT toxin [58], the *E. coli* LT toxin [59], the *Pseudomonas aeruginosa* elastase, exotoxin A, phospholipase C, alkaline phosphatase, and lipases [41,60], as well as other proteins in plant pathogens [17], which are all secreted through their T2S. Some pathogenicity processes facilitated by the T2S include tissue damage, cytotoxicity, adherence, and dissemination [38], and such processes have also been reported in pathogenic *Leptospira* serovars [56]. The role in metabolism and pathogenesis of the T2S of *Leptospira* still waits to be explored.

Some reverse vaccinology strategies have been applied to search for potential immunogenic antigens in *Leptospira* [15]; however, only one study has considered the GspD secretin as a candidate for vaccine trials [16]. In an extensive study of 238 predicted outer membrane or secreted proteins, the authors found GspD as a seroreactive protein. In such a study, GspD was used in a pool of five proteins, including three putative lipoproteins and a TolC-like protein. Despite that all these five proteins were recognized by the sera of vaccinated hamsters, the authors found no protection against leptospirosis in the hamster model [16]. Those findings are in contrast with our results, where the rGspDLC protein mixed with IFA protected up to 75% of hamsters against lethal infection.

Our findings showing that the rGspDLC protein conferred up to 75% protection against disease in the hamster model, the fact that GspD is conserved, abundant [18], and a putative functionally important protein in pathogenic *Leptospira* species, suggest that the T2S GspD secretin of *Leptospira* might well be considered as a potential immunogen to be used for protection against leptospirosis. Further efforts could be directed to demonstrate that GspD as a vaccine protects against other *Leptospira* serovars and species as well as conferring protection in other animal species against leptospirosis, as well as to define the immunogenic region or epitopes of the GspD protein and finally search for the optimization of the expression of such peptides into more efficient hosts other than *E. coli*.

## Figures and Tables

**Table 1:** Treatment protocols applied to hamster groups, protection percentage, infection, and kidney lesion rates.

Group	Treatment	*n*	Survivors (Protection %)	Infection Rates ^a^ PCR Culture		NI ^b^	Kidney Lesions Rates	*p*-Value ^c^	
A	GspDLC + no adjuvant + challenge	8	5 (65.5)	8	3	0	4.875	0.0555	NS ^d^
B	GspDLC + IFA + challenge	8	6 (75)	5	4	3	5	0.0176	
C	Bacterin + challenge	8	8 (100)	1	1	7	3.5	0.0002	
D	IFA + challenge	13	2 (15.3)	13	8	0	7.75	ND ^e^	
E	Saline + challenge	5	0 (0)	5	5	0	8.4	1	NS ^d^

^a^*Leptospira* infection was determined by PCR and culture. ^b^ NI, Not infected hamsters. ^c^ *p*-values are two-sided and were obtained through comparison with group D (Fisher’s exact test). ^d^ NS, Not significant at *p* > 0.05. ^e^ ND, Not determined.
